# Aberrant brain structural–functional connectivity coupling associated with cognitive dysfunction in different cerebral small vessel disease burdens

**DOI:** 10.1111/cns.70005

**Published:** 2024-09-03

**Authors:** Xinyue Zhang, Changhu Liang, Mengmeng Feng, Haotian Xin, Yajie Fu, Yian Gao, Chaofan Sui, Na Wang, Yuanyuan Wang, Nan Zhang, Lingfei Guo, Hongwei Wen

**Affiliations:** ^1^ Key Laboratory of Endocrine Glucose and Lipids Metabolism and Brain Aging, Department of Radiology, Ministry of Education Shandong Provincial Hospital Affiliated to Shandong First Medical University Jinan Shandong China; ^2^ Department of Radiology and Nuclear Medicine, Xuanwu Hospital Capital Medical University Beijing China; ^3^ Shandong Medicine and Health Key Laboratory of Abdominal Medical Imaging, Department of Medical Ultrasound The First Affiliated Hospital of Shandong First Medical University and Shandong Provincial Qianfoshan Hospital Jinan Shandong China; ^4^ School of Medical Imaging Binzhou Medical University Yantai Shandong China; ^5^ Key Laboratory of Cognition and Personality (Ministry of Education), Faculty of Psychology Southwest University Chongqing China

**Keywords:** cerebral small vessel disease burden, cognitive dysfunction, functional efficiency, structural–functional connectivity coupling

## Abstract

**Aims:**

Emerging evidence suggests that cerebral small vessel disease (CSVD) pathology changes brain structural connectivity (SC) and functional connectivity (FC) networks. Although network‐level SC and FC are closely coupled in the healthy population, how SC‐FC coupling correlates with neurocognitive outcomes in patients with different CSVD burdens remains largely unknown.

**Methods:**

Using multimodal MRI, we reconstructed whole‐brain SC and FC networks for 54 patients with severe CSVD burden (CSVD‐s), 106 patients with mild CSVD burden (CSVD‐m), and 79 healthy controls. We then investigated the aberrant SC‐FC coupling and functional network topology in CSVD and their correlations with cognitive dysfunction.

**Results:**

Compared with controls, the CSVD‐m patients showed no significant change in any SC‐FC coupling, but the CSVD‐s patients exhibited significantly decreased whole‐brain (*p* = 0.014), auditory/motor (*p* = 0.033), and limbic modular (*p* = 0.011) SC‐FC coupling. For functional network topology, despite no change in global efficiency, CSVD‐s patients exhibited significantly reduced nodal efficiency of the bilateral amygdala (*p* = 0.024 and 0.035) and heschl gyrus (*p* = 0.001 and 0.005). Notably, for the CSVD‐s patients, whole‐brain SC‐FC coupling showed a significantly positive correlation with MoCA (*r* = 0.327, *p* = 0.020) and SDMT (*r* = 0.373, *p* = 0.008) scores, limbic/subcortical modular SC‐FC coupling showed a negative correlation (*r* = −0.316, *p* = 0.025) with SCWT score, and global/local efficiency (*r* = 0.367, *p* = 0.009 and *r* = 0.353, *p* = 0.012) showed a positive correlation with AVLT score. For the CSVD‐m group, whole‐brain and auditory/motor modular SC‐FC couplings showed significantly positive correlations with SCWT (*r* = 0.217, *p* = 0.028 and *r* = 0.219, *p* = 0.027) and TMT (*r* = 0.324, *p* = 0.001 and *r* = 0.245, *p* = 0.013) scores, and global/local efficiency showed positive correlations with AVLT (*r* = 0.230, *p* = 0.020 and *r* = 0.248, *p* = 0.012) and SDMT (*r* = 0.263, *p* = 0.008 and *r* = 0.263, *p* = 0.007) scores.

**Conclusion:**

Our findings demonstrated that decreased whole‐brain and module‐dependent SC‐FC coupling associated with reduced functional efficiency might underlie more severe burden and worse cognitive decline in CSVD. SC‐FC coupling might serve as a more sensitive neuroimaging biomarker of CSVD burden and provided new insights into the pathophysiologic mechanisms of clinical development of CSVD.

## Graphical abstract

We reconstructed whole‐brain structural connectivity (SC) and functional connectivity (FC) networks for severe cerebral small vessel disease (CSVD) burden patients (CSVD‐s), CSVD mild burden patients (CSVD‐m), and healthy controls. The CSVD‐s group showed considerably decreased SC‐FC coupling in the limbic/subcortical module compared with the CSVD‐m group, while there was no difference in SC‐FC coupling between the CSVD‐m and control groups.

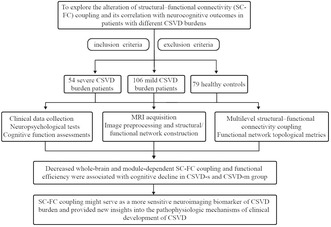

## 1 INTRODUCTION

Cerebral small vessel disease (CSVD) is considered to be an important pathological basis for cognitive dysfunction and vascular dementia, which affects small end arteries, arterioles, venules, and brain capillaries.^1^, ^2^ The terminology used to describe the key imaging features of CSVD was proposed by the Standards for Reporting Vascular Changes on Neuroimaging 1 (STRIVE‐1).^3^ It should be noted that CSVD is a dynamic and global disease^4^; therefore, the overall impact on the brain is better indicated by the total CSVD burden than by any one of the individual imaging indicators. The total CSVD burden is a composite score that is derived from multiple imaging markers, such as white matter hyperintensity (WMH), lacunar, cerebral microbleed (CMB), and enlarged perivascular space (PVS). Studies have validated that a simple CSVD score based on several MRI markers can predict the risk of future dementia,^5^ and CSVD burden is associated with decreased information processing speed and performance on overall cognitive tests.^6^ These findings suggest that grouping studies according to different CSVD burdens is scientifically reliable. Despite this progress in research,^7^ the mechanism of clinical differences shown by different CSVD burdens is still unclear.

Emerging evidence suggests that CSVD pathology changes white matter microstructure, leading to disruptions in brain structural connectivity (SC) networks, which was constructed by diffusion tensor imaging (DTI) and probabilistic diffusion tractography.^8^ Graph theory provides new insights into the underlying mechanisms of diseases by characterizing the internal connections of interregional networks to obtain important topological characteristics of brain networks.^9^ Our previous works have demonstrated that patients with severe CSVD burden suffer greater disruption of structural networks such as global/local efficiency and node efficiency in the brain, mainly in cognitive functional regions.^10^ Prior studies have shown that the severity or total burden of CSVD is related to lower brain network density, connection strength and network efficiency and poorer cognitive performance.^11^ Baseline network efficiency predicts mortality risk, which can reflect overall brain health in CSVD.^12^ Previous studies have proven that lower global efficiency involving primarily frontal tracts is related to poorer gait performance, and an advanced cognitive process including shorter strides and slower gait speeds^13^ and reduced neural network efficiency, especially in the reward network, is associated with increased apathy symptoms in CSVD patients.^14^ Recently, resting‐state functional MRI (rs‐fMRI) has been widely used to construct functional networks, and studies have shown that multiple functional networks composed of distributed brain regions that share synchronous spontaneous activity coexist in the brain.^15^ For functional connectivity (FC) in CSVD functional networks within or between the dorsal attention network (DAN), default mode network (DMN), and frontoparietal control network (FPCN), which are mainly responsible for attention and executive function, are impaired.^16^ Furthermore, graph theory analysis was used to analyze CSVD patients with cerebral microbleeds, which showed significantly increased shortest path length and decreased global efficiency, local efficiency, and clustering coefficient.^17^ Studies on Alzheimer's disease have found differences in FC abnormalities between CSVD and non‐CSVD individuals, influenced by CSVD burden and neuropathology.^18^ With the increase in disease burden, the decrease in FC is more serious.

Despite these advances, structural and functional connections are two inseparable concepts, and the effects of SC on FC networks remain largely unknown. The focus of research on the whole brain network should be attributed to combining them to explore their interaction, rather than separating them completely. In healthy individuals, the structural and functional networks are tightly coupled and change in their similarity with development.^19^ Aberrant SC‐FC coupling has been observed in a series of neuropsychiatric conditions, among which multiple clinical symptoms of CSVD, such as behavioral symptoms and motor disorders. Wang et al. recently found that mild to moderate cognitive impairment without dementia has higher structure–function connectome correlations than controls, which implies poor executive function and verbal memory.^20^ Zhang et al. revealed that the intensity of structure–function connectivity network coupling is reduced in patients with ischemic stroke, and the degree of coupling is correlated with the Fugl‐Meyer Assessment (FMA) score.^21^ In recent studies on CSVD, Tay et al. demonstrated that decreased SC‐FC coupling was observed in CSVD and that decreased coupling in cognitive control networks was related to PS, apathy, and general cognition in both longitudinal and cross‐sectional studies.^22^ However, the limitation of the above studies is that the effects of different burdens of CSVD on SC‐FC coupling and its cognition were not explored. Furthermore, impaired FC network efficiency has been related to a higher burden of CSVD pathology and cognitive impairment.^17^, ^23^ It is therefore possible that the altered SC‐FC coupling and functional efficiency of CSVD with different burdens and its relationship to cognitive outcomes has yet to be investigated.

In this study, we aimed to construct whole‐brain SC and FC networks in different CSVD burdens patients and healthy controls using multimodal MRI. We then investigated how subject‐level SC‐FC coupling and functional network changes correlated with five prominent neurocognitive metrics of CSVD. We hypothesized that SC‐FC coupling and functional efficiency would decrease with the increased burden of CSVD and would be associated with more severe cognitive decline. In addition, we attempted to further explore whether the cognitive effects of SC‐FC decoupling are limited to specific functional modules of the brain.

## 2 MATERIALS AND METHODS

### 2.1 Subjects

Shandong Provincial Hospital, which is affiliated with Shandong First Medical University, granted approval for the cross‐sectional study through its institutional review board. A total of 54 participants with severe CSVD burden (CSVD‐s; age: 63.89 ± 6.12 years; 34 males) and 106 participants with mild CSVD burden (CSVD‐m; age: 61.58 ± 7.65 years; 58 males) were included between December 2018 and January 2022. We also incorporated 79 healthy volunteers (age: 60.48 ± 9.91 years; 35 males) who were matched for gender, age, and education into our study. Each participant willingly provided an informed permission form prior to commencing the study.

In accordance with prevailing MRI consensus standards, CSVD patients had to meet certain inclusion criteria, which included a diagnosis of lacunes of presumed vascular origin, WMH of presumed vascular origin, enlarged PVS, CMBs, recent small subcortical infarct, and brain atrophy.^3^ The “total SVD burden,”^24^ a practical ordinal scale of 0–4 that is based on the initial four of the previously addressed MRI markers of CSVD, was used to evaluate the severity of CSVD: one point was given if there was at least one lacune present; one point was given if there was early confluent deep WMH (Fazekas score^25^ 2 or 3) or irregular periventricular WMH extending into the deep white matter (Fazekas score 3); one point was given if there was moderate to severe (grades 2 and 3) PVS in the basal ganglia; and one point was given if at least one CMB was present. Subjects who scored between 0 and 1 were allocated to the CSVD‐m group, and those who scored between 2 and 4 were allocated to the CSVD‐s group.^10^

The study's exclusion criteria consisted of six factors: (1) a history of epilepsy, brain trauma, stroke and brain tumors; (2) a history of substance abuse or alcoholism; (3) a history of thrombolysis treatment; (4) heart, kidney and liver injury; (5) acute and severe complications of type 2 diabetes and hypertension; and (6) a history of major neurologic or psychiatric illnesses.

### 2.2 MRI acquisition

A 3.0‐Tesla MR system (Siemens Healthcare, Erlangen, Germany) with a 32‐channel head coil for signal reception was utilized to obtain MRI scans. T1‐weighted images were obtained through a magnetization‐prepared rapid gradient echo (MPRAGE) sequence: field of view (FOV) = 240 × 240 mm^2^; matrix size = 256 × 256; inversion time (TI) = 900 ms; repetition time (TR)/echo time (TE) = 7.3/2.4 ms; slice thickness = 1 mm, 192 slices, and slice thickness = 0.9 mm with no gap; flip angle = 9°. DTI data were acquired using a simultaneous multislice (SMS) accelerated single‐shot echo planar imaging (EPI) sequence with the following parameters: TR/TE = 3000/110 ms; FOV = 220 × 220 mm; matrix = 110 × 110; slice thickness = 2.2 mm without gap; 60 axial slices; 30 diffusion gradient directions (*b* = 1700 s/mm^2^) plus one b_0_ reference image. Using a gradient‐echo echo‐planar imaging (GE‐EPI) sequence, resting‐state blood oxygen level dependent (BOLD) fMRI data were obtained: FOV = 240 × 240 mm; TR/TE = 1500/30 ms; matrix size = 64 × 64; slice gap = 1 mm, slice thickness = 3 mm; 50 axial slices; 200 volumes.

### 2.3 Cognitive assessments

The one‐page, 30‐point Montreal Cognitive Assessment (MoCA) Beijing version (www.mocatest.org) was given in 10 min.^26^ For illiterate subjects, 13/14 was the best cutoff for identifying cognitive impairment, followed by 19/20 for people with 1–6 years of education and 24/25 for people with 7 years or more.^27^ Additionally, a number of executive functions were evaluated, such as working memory, inhibition, and adaptability. These tests included, specifically, the following: the trail‐making test (TMT) for motor coordination, evaluating attention, visual searching, and information processing speed^28^ and the Stroop color‐word test (SCWT)^29^; the symbol digit modalities test (SDMT)^30^; and the Rey auditory verbal learning test (AVLT).^31^ We refer to international standards for testing and scoring (for details, see Supplementary Methods). The experimenter was not aware of the subject group and professionally trained and qualified.

### 2.4 Image preprocessing and structural/functional network construction

**FIGURE 1:**
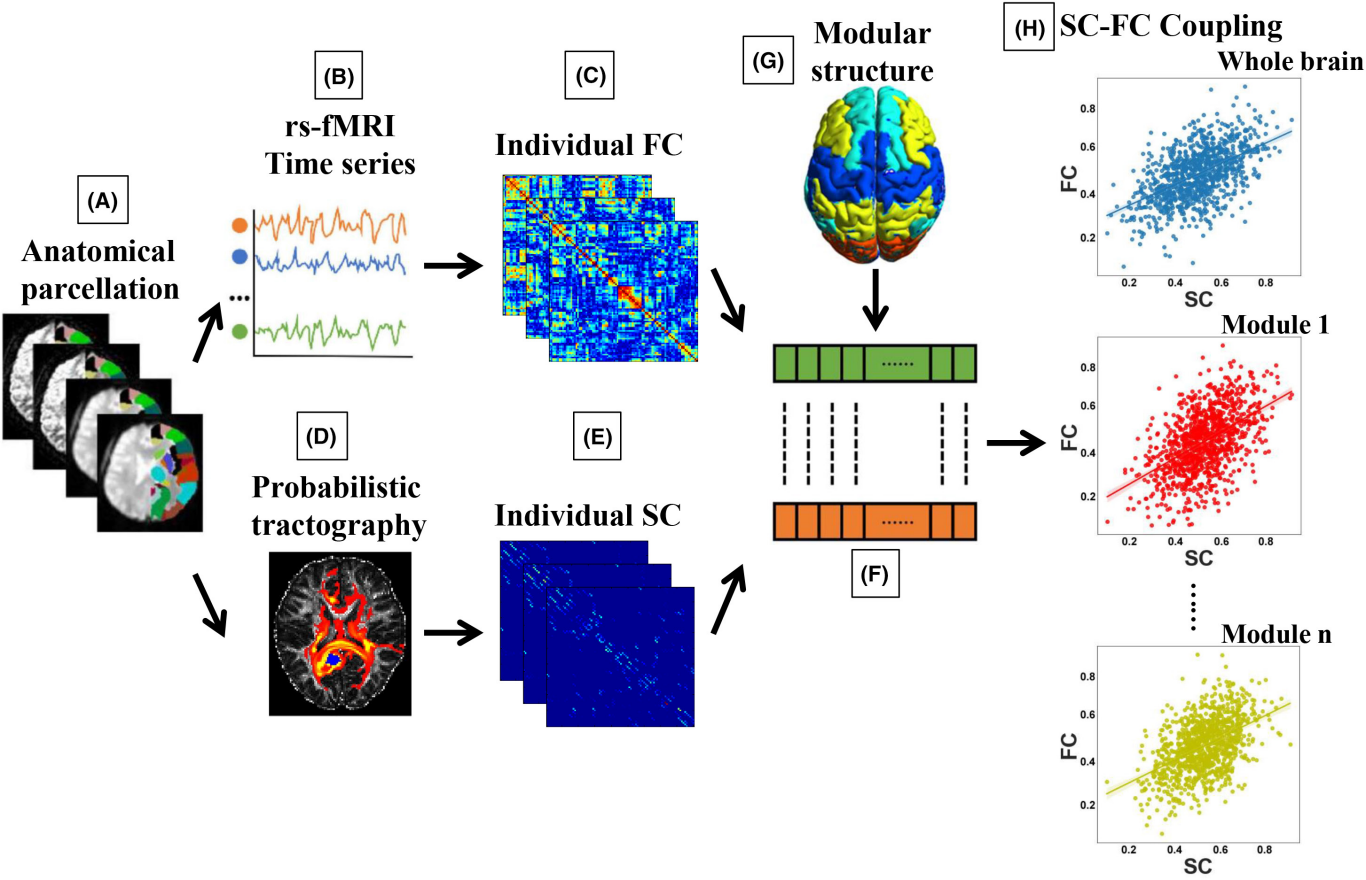
Flowchart of structural–functional coupling analysis. (A) The brain was divided into 90 separate brain regions using an automated anatomical labeling (AAL) template. These regions also operated as nodes of the individual brain functional connectivity (FC) and structural connectivity (SC) networks. (B) Rs‐fMRI was used to generate ROI‐wise bold time series. (C) The FC matrix was established by determining the absolute Pearson correlation coefficient of the interregional time series. (D) Whole‐brain white matter pathways were constructed by diffusion MRI probabilistic tractography. (E) The edge weight of the SC matrix was established by calculating the interregional connectivity probability. (F) The nonzero FC in sparse functional networks and the corresponding SC were transformed into a vector. (G) We used a well‐known predefined modular division^34^ for subsequent calculation of modular SC‐FC coupling. (H) Modular SC–FC coupling was determined by calculating the Pearson correlation coefficient between the matching FC and SC vectors in each module.

Following our previous studies,^32^, ^33^ we preprocessed the rs‐fMRI data and constructed FC network using the Data Processing & Analysis for Brain Imaging toolbox (DPABI v6.0, http://rfmri.org/dpabi). In detail (Figure 1A–C), the main processes involved (1) eliminating the first 10 time points, (2) correcting slice timing, and (3) correcting head motion. Above all, no participant was excluded due to the following exclusion criteria: maximum head motion of 3.0 mm and 3.0 degrees and mean framewise displacement (FD) >0.2 mm^35^; (4) regressing out nuisance covariates, such as the 24 rigid body motion parameters and signals from cerebrospinal fluid (CSF) and white matter (WM); (5) normalizing to Montreal Neurological Institute (MNI) standard space at 3‐mm isotropic voxel resolution using DARTEL^36^; (6) spatially smoothing using a Gaussian kernel (full width at half maximum = 4 mm); and (7) bandpass filtering (0.01–0.08 Hz); (8) Utilizing the automated anatomical labeling (AAL) template to extract the averaged rs‐fMRI time patterns in 90 different brain regions (Table S1). Pearson's correlation coefficients were then computed between the averaged time courses of each pair of regions, resulting in a 90 × 90 symmetric FC networks.

Using the FMRIB Software Library (FSL v5.0, http://www.fmrib.ox.ac.uk/fsl), we conducted DTI image preprocessing and brain SC network construction in accordance with our previous study.^8^ Affine alignment of each diffusion‐weighted image was applied to the b0 image for each participant to adjust motion artifacts and eddy current distortions by using FSL's eddy‐correction tool. Afterwards, to eliminate nonbrain voxels from further analysis, brain masks were generated from each b0 image provided by FMRIB's brain extraction tool (BET v2.1). Next, the FMRIB diffusion toolbox (FDT v3.0) was used to estimate the diffusion tensor model at each voxel, and a two‐tensor model was used to predict the probability distribution of fiber orientations from each voxel.^37^ Probabilistic tractography was performed for each subject (Figure 1D) to define the SC between brain regions. The network edges w_ij_ were computed as the probability of connectivity from seed region i to given region j, resulting in a 90 × 90 SC network/matrix (Figure 1E).

### 2.5 Multilevel structural–functional connectivity coupling

To quantitatively analyze multilevel SC‐FC coupling, we performed a correlation analysis between the strength of the functional connections and their structural counterparts throughout the whole brain and each functional module.^38^ To eliminate the bias of a single threshold selection on sparse brain networks, as in our previous study,^32^ a series of sparsity thresholds of FC (from 8% to 60% with an interval of 2%) was used to generate sparse functional networks with weak edges substituted by zeros. The nonzero FC in sparse functional networks was extracted and reshaped to a vector and the counterpart SC was also reshaped to another vector. The Pearson correlation coefficients between the nonzero FC and the counterpart SC were then used to calculate the SC‐FC coupling.^39^ This approach resulted in one whole‐brain SC‐FC coupling for each participant at each sparsity threshold of FC (Figure 1F).

Additionally, to ensure the comparability between groups of modular SC‐FC coupling, we should keep the same functional modular structure for all groups. Therefore, we applied a well‐known predefined functional modular division^34^ on the FC network for all groups, in which the resting‐state brain network was modularly configured and optimally organized into five functional modules: auditory/motor (20 regions), vision (14 regions), attention (18 regions), default‐mode network (18 regions), and limbic/subcortical (20 regions) systems (Table S1). Then, modular SC‐FC coupling was calculated by selecting nonzero FC within each module and correlating it with their SC counterparts (Figure 1G,H).

### 2.6 Functional network topological metrics

For each sparsity threshold of FC networks, we investigated the global and regional topological metrics (for detailed definitions, see Supplementary Methods) of FC networks using a graph theoretical network analysis toolbox (GRETNA, http://www.nitrc.org/projects/gretna/).^40^ Three global topological measures including shortest path length (*L*_p_), global efficiency (*E*_glob_), and local efficiency (*E*_loc_) were calculated. For nodal measures, we considered the nodal efficiency (*E*_nodal_). Notably, to give a summary metric independent of single threshold selection, the area under the curve (AUC) was also calculated for all topological metrics.^32^ Not only at each sparsity threshold, the integrated AUC values of SC‐FC coupling and functional topological metrics were also compared between groups.

### 2.7 Between‐group statistical comparison and correlation analysis

The Kolmogorov–Smirnov (K–S) test was used to perform the normality test. Then, one‐way analysis of variance (ANOVA) and least significant difference (LSD) pairwise multiple comparison tests were used to evaluate differences in head motion, age, education, and cognitive test scores within the three groups, and the non‐parametric chi‐square test was utilized to analyze the counting data such as sex ratio, history of hypertension, diabetes mellitus, and hyperlipidemia. According to the findings of our recent studies and clinical experience,^17^, ^41^ head motion, age, sex, and education were potential confounders for investigating between‐group differences in neuroimaging metrics. Although the subjects had a history of hypertension, their blood pressure was controlled within the normal range at the time of MRI examination. The bias caused by it did not have a significant effect on the results. One‐way analysis of covariance (ANCOVA), which controls for head motion, age, sex, and education as covariates, was used to examine differences between the three groups for SC‐FC coupling and functional network topological metrics. Once significant intergroup variations in any network metrics were identified, we used SPSS v24.0 software to calculate the partial correlation coefficients between the network metrics and cognitive parameters for each group, controlling for head motion, age, sex, and education as covariates. All analyses were conducted with a significance level of *p* < 0.05.

## 3 RESULTS

### 3.1 Demographic and clinical characteristics of the subjects

Table 1 provides an overview of each group's demographic and cognitive characteristics. Head motion, age, education, and cognitive parameters all follow a normal distribution (shown in Table S2), except for educational level which follows an approximate normal distribution (shown in Figure S2). The CSVD‐s group had significantly lower MoCA, AVLT, and SDMT scores and significantly higher SCWT and TMT scores than the CSVD‐m and control groups, except there was no significant difference in MoCA score between the CSVD‐s and CSVD‐m groups. Furthermore, compared to the control group, the CSVD‐m group had significantly lower SDMT scores. Head motion, age, sex, education level, diabetes mellitus, hyperlipidemia, and smoking parameters did not significantly differ between the groups and history of hypertension had significant differences among the three groups.

**TABLE 1:** Demographic and clinical characteristics of CSVD patients and controls.

Characteristic	CSVD‐s	CSVD‐m	HC	*p* value (ANOVA/*χ* ^2^)	*p* value (post hoc)		
					CSVD‐s vs. HC	CSVD‐s vs. CSVD‐m	CSVD‐m vs. HC
Gender	34 M/20 F	58 M/48 F	35 M/44 F	0.097^*χ*2^	‐	‐	‐
Age (years)	63.89 ± 6.12	61.58 ± 7.65	60.48 ± 9.91	0.061^a^	‐	‐	‐
History of hypertension	40 (74.1%)	52 (49.1%)	27 (34.2%)	<0.001^*χ*2^			
Diabetes mellitus	24 (44.4%)	49 (46.2%)	29 (36.7%)	0.414^*χ*2^			
Hyperlipidemia	27 (50.0%)	41 (38.7%)	31 (39.2%)	0.346^*χ*2^			
Smoking	16 (29.6%)	26 (24.5%)	22 (27.8%)	0.762^*χ*2^			
Education (years)	11.22 ± 3.31	12.12 ± 3.19	12.66 ± 3.49	0.052^a^	‐	‐	‐
MoCA	24.39 ± 2.89	25.38 ± 3.59	26.19 ± 3.76	0.017^a^	0.004	0.096	0.121
AVLT	54.96 ± 13.29	61.25 ± 12.55	64.00 ± 12.36	<0.001^a^	<0.001	0.004	0.146
SCWT	179.8 ± 58.93	146.21 ± 44.79	133.53 ± 37.93	<0.001^a^	<0.001	<0.001	0.068
SDMT	26.18 ± 11.85	32.18 ± 12.43	40.42 ± 14.33	<0.001^a^	<0.001	0.007	<0.001
TMT(B‐A)	165.51 ± 93.55	124.9 ± 107.68	110.64 ± 96.73	0.010^a^	0.003	0.020	0.349
FD_Jenkinson	0.13 ± 0.04	0.12 ± 0.04	0.11 ± 0.04	0.171^a^	‐	‐	‐
Lacunes (1 point)	23 (42.6%)	2 (1.9%)	‐	<0.001^*χ*2^			
WMHs (1 point)	46 (85.2%)	21 (19.8%)	‐	<0.001^*χ*2^			
PVSs (1 point)	48 (88.9%)	34 (32.1%)	‐	<0.001^*χ*2^			
CMBs (1 point)	35 (64.8%)	13 (12.3%)	‐	<0.001^*χ*2^			

*Note*: History of hypertension: number and proportion of subjects with a history of hypertension whose blood pressure was stable in the normal range at the time of the MRI examination. FD_Jenkinson: framewise displacement.^35^

Abbreviations: AVLT, sum of the rey auditory verbal learning test (N1‐7); CSVD‐m, Mild CSVD burden group (score ≤1); CSVD‐s, severe CSVD burden group (score ≥2); HC, healthy control; MoCA, Montreal Cognitive Assessment; SCWT, sum of stroop color‐word test (stroop 1–3); SDMT, symbol digit modalities test; TMT (B‐A), the difference between TMT‐B and TMT‐A; TMT, the trail‐making test.

^a^ ANOVA test^*χ*2^: chi‐square test.

### 3.2 Disrupted SC‐FC coupling and functional global efficiency in CSVD

**FIGURE 2:**
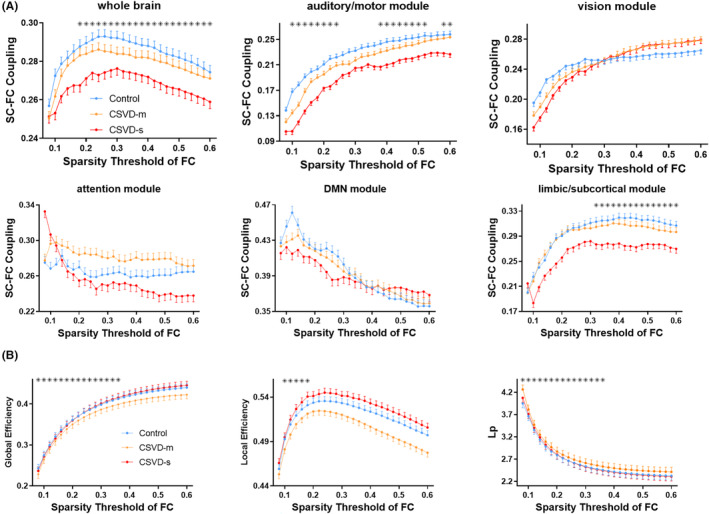
Group comparisons of SC‐FC coupling (A) and efficiency metrics (B) across sparsity thresholds. The dotted line chart presents the mean values of each statistic for each group, with the error bar represents the standard deviation, and the black asterisk above indicates that the corresponding statistical data below shows a significant difference (*p* < 0.05, ANCOVA) among three groups under the corresponding sparsity threshold of FC. CSVD‐s, severe CSVD burden group; CSVD‐m, mild CSVD burden group; SC‐FC, structural connectivity and functional connectivity; Lp, shortest path length.

The SC‐FC coupling within whole brain and each functional module exhibited different patterns between groups. Briefly, the CSVD‐s group showed significantly decreased SC‐FC coupling within the whole brain, sensory/motor, and limbic/subcortical functional modules over a broad range of sparsity thresholds of FC (Figure 2A) compared with the control group. The AUC values of SC‐FC coupling were also significantly decreased in those modules for the CSVD‐s group (Table 2), indicating the consistency and robustness of significant alterations across different sparsity thresholds. In addition, the CSVD‐s group also showed considerably decreased SC‐FC coupling in the limbic/subcortical module compared with the CSVD‐m group, while there was no difference in SC‐FC coupling between the CSVD‐m and control groups.

**TABLE 2:** Group comparisons of SC‐FC coupling and functional topological metrics.

Metrics (AUC value)	Module	Groups			*p* value (ANCOVA)	*p* value (post hoc)		
		CSVD‐s	CSVD‐m	HC		CSVD‐s vs. HC	CSVD‐s vs. CSVD‐m	CSVD‐m vs. HC
SC‐FC coupling and global topological metrics								
Coupling_whole brain_	‐	0.268 ± 0.032	0.278 ± 0.034	0.284 ± 0.03	0.014	0.004	N.S.	N.S.
Coupling_aufitory/motor_	‐	0.192 ± 0.088	0.214 ± 0.081	0.228 ± 0.065	0.033	0.009	N.S.	N.S.
Coupling_vision_	‐	0.247 ± 0.079	0.250 ± 0.081	0.249 ± 0.092	0.977	‐	‐	‐
Coupling_attention_	‐	0.255 ± 0.082	0.281 ± 0.106	0.264 ± 0.099	0.242	‐	‐	‐
Coupling_DMN_	‐	0.389 ± 0.072	0.393 ± 0.069	0.395 ± 0.055	0.878	‐	‐	‐
Coupling_subcortical_	‐	0.262 ± 0.072	0.291 ± 0.072	0.297 ± 0.058	0.011	0.004	0.013	N.S.
*E*_glob_ (×e^−1^)	‐	3.906 ± 0.495	3.754 ± 0.378	3.888 ± 0.437	0.039	N.S.	0.034	0.035
*E*_loc_ (×e^−1^)	‐	5.264 ± 0.742	5.031 ± 0.615	5.178 ± 0.694	0.092	‐	‐	‐
*L*_p_	‐	2.668 ± 0.290	2.760 ± 0.257	2.665 ± 0.272	0.030	N.S.	0.042	0.019
Regional topological metrics: *E*_nodal_ (×e^−1^)								
ORBmid.L	Attention	3.828 ± 0.674	3.562 ± 0.579	3.643 ± 0.622	0.037	N.S.	0.010	N.S.
IPL.R	Attention	3.705 ± 0.858	3.422 ± 0.547	3.608 ± 0.703	0.030	N.S.	0.014	N.S.
ANG.R	Attention	3.625 ± 0.903	3.379 ± 0.544	3.587 ± 0.628	0.037	N.S.	0.028	0.038
SFGmed.L	DMN	4.076 ± 0.684	3.763 ± 0.578	3.836 ± 0.572	0.008	0.025	0.002	N.S.
SFGmed.R	DMN	3.990 ± 0.698	3.659 ± 0.612	3.762 ± 0.520	0.005	0.033	0.001	N.S.
PCUN.R	DMN	4.194 ± 0.838	3.866 ± 0.663	3.929 ± 0.798	0.031	0.047	0.010	N.S.
AMYG.L	Limbic	3.089 ± 0.976	3.265 ± 0.811	3.491 ± 0.787	0.024	0.007	N.S.	N.S.
AMYG.R	Limbic	3.159 ± 0.966	3.337 ± 0.801	3.533 ± 0.740	0.035	0.011	N.S.	N.S.
HES.L	Auditory	3.251 ± 1.051	3.555 ± 0.861	3.851 ± 0.812	0.001	<0.001	0.043	0.026
HES.R	Auditory	3.400 ± 0.840	3.639 ± 0.835	3.875 ± 0.788	0.005	0.001	N.S.	N.S

*Note*: *E*_nodal_ is a representation of the AUC values (mean ± standard deviation) of the nodal efficiency for each group. The modular division of brain regions was based on a previous study.^34^ For abbreviations of region, refer to Table S1.

Abbreviations: AUC, area under the ROC curve; CSVD‐m, mild CSVD burden group (score ≤1); CSVD‐s, severe CSVD burden group (score ≥2); DMN, default mode network; HC, healthy controls; NS, not significant.

For global topological metrics of functional networks, the CSVD‐m group demonstrated significantly decreased *E*_glob_/*E*_loc_ and increased *L*_p_ over a wide range of thresholds (Figure 2B), as well as significantly decreased AUC values of E_glob_ and increased L_p_ compared with the control group. No significant difference was observed in any topological metrics between CSVD‐s and control groups (Table 2).

### 3.3 Disrupted nodal efficiency of functional networks among groups

**FIGURE 3:**
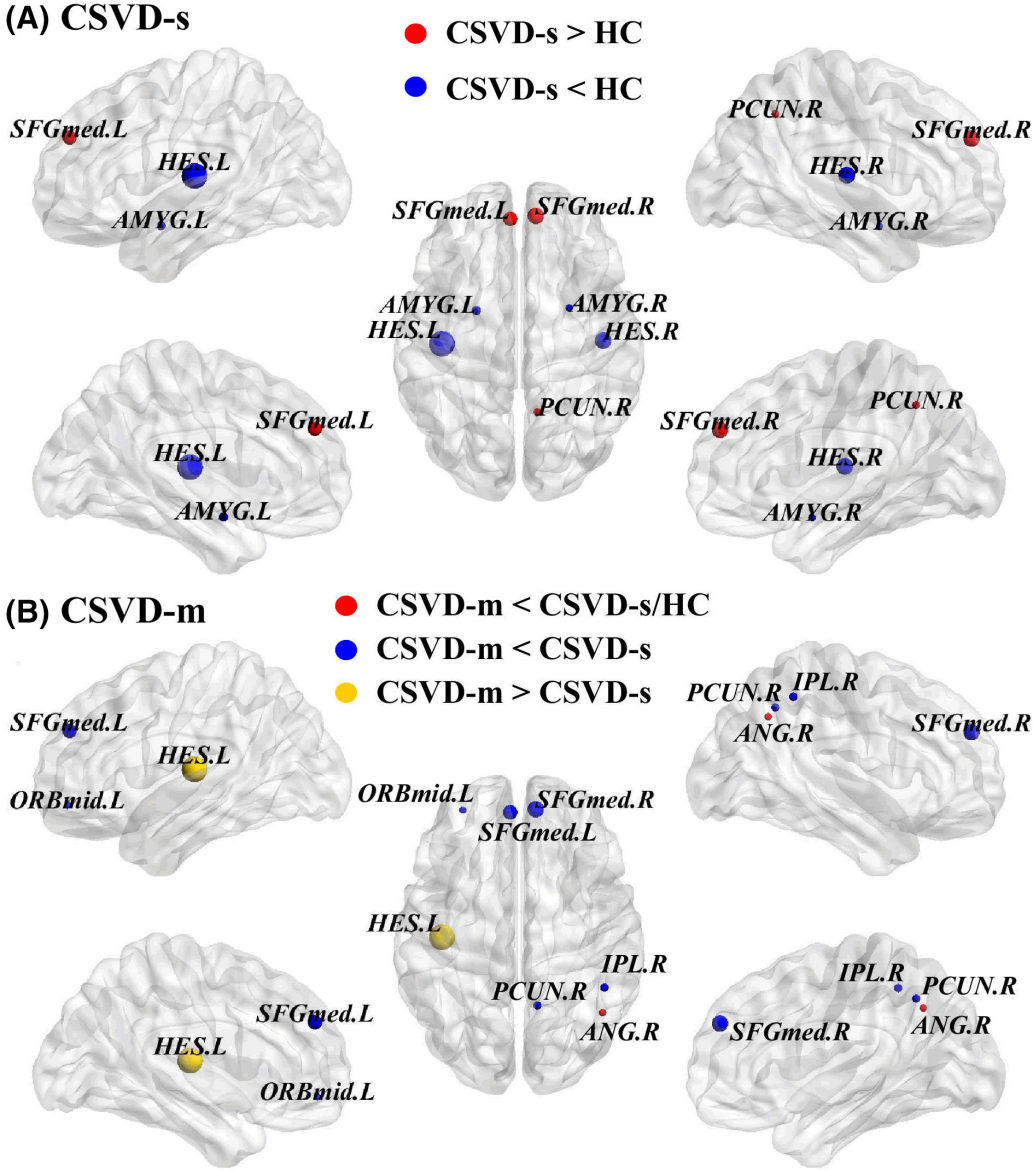
Nodes with altered efficiency among groups. (A) The CSVD‐s and (B) CSVD‐m patients represented significantly altered nodal efficiency, and the scaled node sizes represented the *F* values in the ANCOVA test. *BrainNet Viewer* software (http://www.nitrc.org/projects/bnv/) was used to view the brain graphs. For the abbreviations of nodes, see Table S1.

Among the three groups, 10 brain regions showed significantly altered nodal efficiency (Table 2). Briefly, compared with the controls, CSVD‐s patients demonstrated significantly reduced nodal efficiency in the bilateral amygdala and heschl gyrus within auditory/motor and limbic functional modules with SC‐FC coupling declines, as well as increased nodal efficiency in the bilateral medial superior frontal gyrus (SFGmed) and right precuneus within the default mode network (DMN) with no SC‐FC coupling change. In addition, CSVD‐m patients demonstrated considerably decreased nodal efficiency in the bilateral SFGmed, right precuneus, angular gyrus, inferior parietal gyrus, and left orbital middle frontal gyrus (ORBmid), involving attention and DMN modules with no SC‐FC coupling change compared to CSVD‐s patients. Meanwhile, the CSVD‐m patients also exhibited significantly decreased nodal efficiency in the right angular gyrus and left heschl gyrus (Table 2, Figure 3).

### 3.4 Altered SC‐FC coupling and network efficiency related to cognitive performance in CSVD

As the coupling/efficiency metrics and cognitive scores all follow normal distribution (shown in Tables S2 and S3), the partial correlations between altered coupling/efficiency metrics and cognitive scores were calculated after controlling head motion, age, sex, and education as covariates. We discovered widespread significant associations (*p* < 0.05, FDR corrected) in both the CSVD‐s and CSVD‐m groups (detailed shown in Figure S3A,B), while the control group showed no significant association (Figure S3C).

**FIGURE 4:**
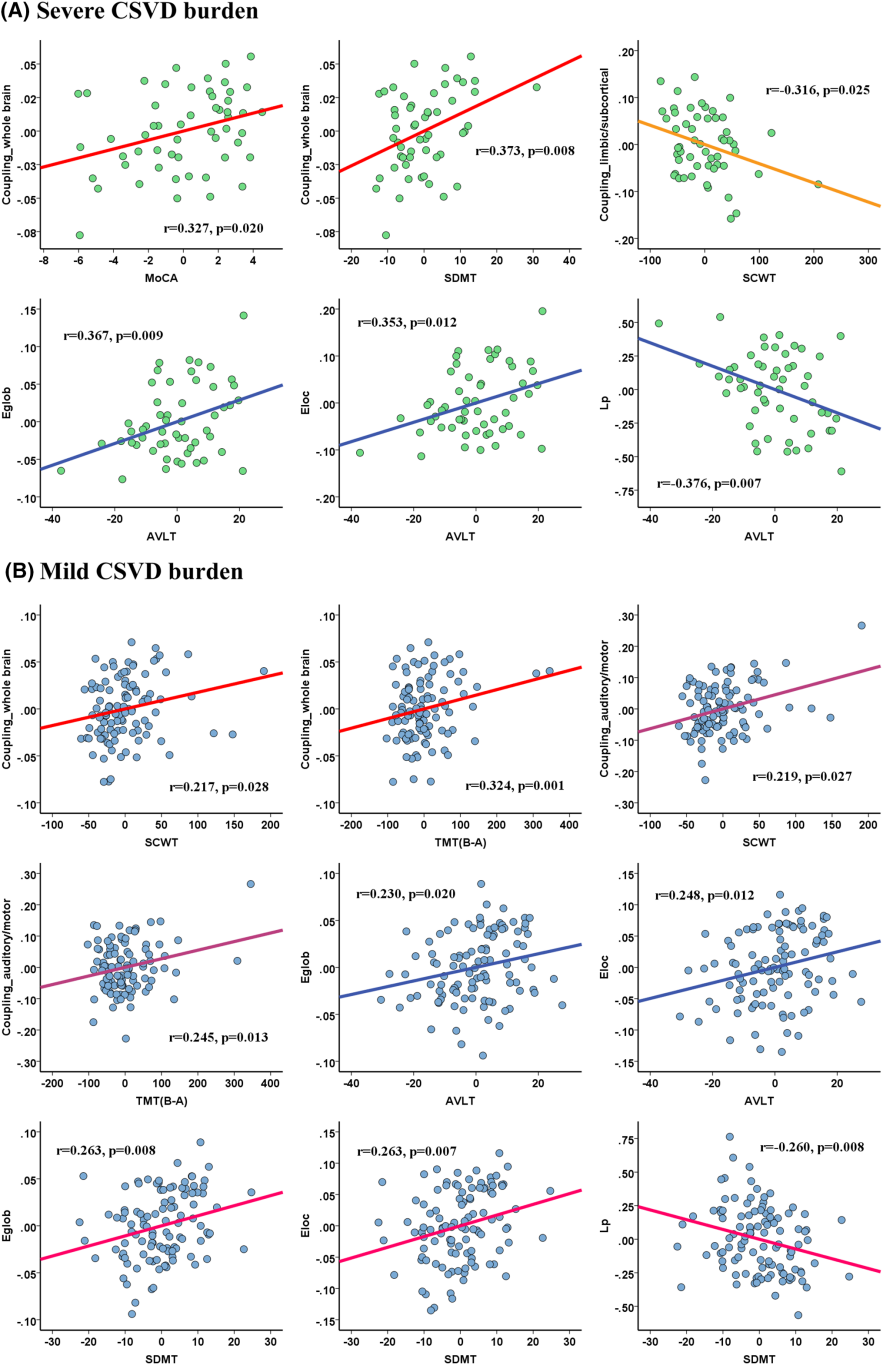
Correlations between the coupling/efficiency metrics and cognitive scores in the severe CSVD burden (A) and mild CSVD burden group (B). Notably, the coordinate values of both the *x* axis (cognitive score) and *y* axis (network metric) represent the residual values after controlling age, sex, education, and head motion as covariates.

Specifically, for the CSVD‐s group, whole‐brain SC‐FC coupling showed a significantly positive correlation with MoCA (*r* = 0.327, *p* = 0.020) and SDMT (*r* = 0.373, *p* = 0.008) scores, limbic/subcortical modular SC‐FC coupling showed a negative correlation (*r* = −0.316, *p* = 0.025) with SCWT score, and global/local efficiency (*r* = 0.367, *p* = 0.009 and *r* = 0.353, *p* = 0.012) showed a positive correlation with AVLT score (Figure 4A). For the CSVD‐m group, whole‐brain and auditory/motor modular SC‐FC couplings showed significantly positive correlations with SCWT (*r* = 0.217, *p* = 0.028 and *r* = 0.219, *p* = 0.027) and TMT (*r* = 0.324, *p* = 0.001 and *r* = 0.245, *p* = 0.013) scores, and global/local efficiency showed positively correlations with AVLT (*r* = 0.230, *p* = 0.020 and *r* = 0.248, *p* = 0.012) and SDMT (*r* = 0.263, *p* = 0.008 and *r* = 0.263, *p* = 0.007) scores (Figure 4B).

## 4 DISCUSSION

The current study reconstructed whole‐brain SC and FC networks using multimodal MRI and then investigated the aberrant SC‐FC coupling and functional network topology for CSVD patients with severe or mild burden. We revealed three main findings: (i) the CSVD‐s patients exhibited significantly decreased SC‐FC coupling within the whole brain, auditory/motor, and limbic/subcortical functional modules compared with controls; (ii) for functional network topology, despite no change in global efficiency, CSVD‐s patients exhibited markedly reduced nodal efficiency in the bilateral amygdala and heschl gyrus within auditory/motor and limbic/subcortical functional modules with SC‐FC coupling declines; (iii) whole brain and modular SC‐FC coupling and functional global/local efficiency were significantly associated with different cognitive testing scores for CSVD‐s and CSVD‐m patients, respectively. Given the vital role of mature brain network topology in information processing and integration,^42^ our results provide new insights into how disruption of SC‐FC coupling and functional network topology relates to global cognitive deficits in the CSVD brain.

While people often focus on the interactions between specific brain regions related to cognitive tasks, their relationship to the underlying anatomical connections and physiological processes tends to be overlooked. Emerging evidence demonstrates that structural and functional networks do not exist independently but interact with each other. Although resting‐state functional connections often exist between regions with no direct structural connection, their persistence and strength are limited by the large‐scale anatomy of the human cerebral cortex.^43^ SC provides the framework for FC and imposes constraints on FC. Strong patterns of functional connectivity are typically seen in brain regions that are structurally closely connected.^44^ In contrast, FC affects SC through plasticity mechanisms.^45^ When the normal structure in the brain is damaged, the structural constraints on function are reduced, which leads to a dynamical enhancement of the functional network, manifested as an anomalous coupling of SC‐FC. Importantly, the results obtained in our study also confirmed previous research on the structure–function coupling of CSVD.^22^ One difference is that our study not only explored the large‐scale network of the whole brain but also divided it into different functional modules to explore the possible stronger correlation between SC‐FC coupling in fine‐grained networks and cognitive performance, and another is the detailed study of the disruption of brain networks with different burdens of CSVD.

Mechanistically, demyelination of white matter microstructure, gliosis, and severe fiber loss may impair the integrity of white matter, reflecting changes in structural network connectivity in CSVD.^46^, ^47^ Our recent study revealed that CSVD‐s patients demonstrated substantially decreased local efficiency, indicating that the information transmission capacity between adjacent brain regions significantly declined.^10^ The change in signal molecules such as neurotransmitter receptors in the cerebral cortex may cause phase dissynchronization between neurons, resulting in the imbalance of functional integration and separation of brain networks.^48^, ^49^ Further studies are needed to investigate the pathophysiological mechanisms. The alteration of the coupling value at the whole‐brain level may be due to interactions between the aforementioned biological underpinnings. A significant reduction in SC‐FC coupling in CSVD‐s patients may indicate a disruption of coherence between structural and functional connections. However, the lack of significant changes in coupling in the CSVD‐m group may be due to a compensatory mechanism between structural and functional connectivity, that is, the autoregulatory function of the brain in the early stages of CSVD. Research has argued that the brain has the ability to tolerate a certain degree of brain damage, and the brain's reserve capacity plays a role after the occurrence of CSVD, which can actively compensate for the clinical deterioration of pathology.^50^ Alteration of structural and functional network efficiency is also considered as a form of compensatory mechanism.^51^

A series of convergent studies have shown that spontaneous brain functional networks have an internally cohesive modular structure, thus dividing complex resting‐state functional MRI networks into motor, visual, attentional, auditory, subcortical, and “default” networks.^34^ Furthermore, the structures consistently match their functions, especially with greater accuracy in vision, somatosensory, and DMN.^19^ Several core network connectivity areas, such as the limbic/subcortical module, are crucial to the coordination of the whole network information flow, and their damage has a significant impact on the integrity and stability of multiple brain function systems, such as working memory and executive function. Therefore, it can be speculated that the decreased coupling in CSVD‐s patients is related to cognitive deficits such as motor, memory, and executive dysfunction. Moreover, the above results also illustrate that the burden score‐based subject grouping strategy is valid and suitable for further exploration of the development process and imaging abnormalities of CSVD. Intriguingly, no significant coupling changes were found in the DMN, one of the intrinsic connectivity networks (ICAs) that has been shown to be associated with cognitive deficits in neurological diseases. One possible explanation is brain plasticity, the ability of the brain to acquire new skills and adapt to new environments by reorganizing its structural and functional connectivity in response to internal and external stimuli.^52^ Studies have shown that the connectivity in certain brain regions of the DMN basically recovers 3 months after stroke, which is significantly related to cognitive function recovery.^53^ It is therefore reasonable to infer that lower correlations between structural and functional networks in the DMN module may represent functional reorganization along indirect white matter pathways. Reduced coupling in auditory/motor and limbic/subcortical functional modules represents damage to their structural or functional connectivity, and these connections are not compensated.

In the study of functional network topology, our results indicated that CSVD‐m and CSVD‐s patients showed discrepant trends in global efficiency and nodal efficiency and that the topological organization of FC networks of both was disrupted. To our knowledge, it is relatively scarce to study the global and nodal efficiency variation trends of different burden groups. Specifically, the functional network is able to perform adaptive processes as the disease burden increases. The self‐optimization of the brain network in CSVD‐s patients due to the excessive burden can improve the efficiency of functional network topology organization to meet the needs of normal function operation. In the CSVD‐s group, the four brain regions (bilateral amygdala and heschl gyrus) with decreased nodal efficiency were located in the functional module with reduced SC‐FC coupling. Moreover, these results also showed that the information transmission between remote areas of CSVD‐m patients was reduced, which was mainly related to impaired remote connections. Compared with the CSVD‐s group, the nodal efficiency of the CSVD‐m group was also decreased, but the consistency of the above result did not appear. Thus, we speculated that the functional efficiency of CSVD‐m patients was decreased globally, while in the severe burden group, the functional efficiency of some brain regions in modules with decreased SC‐FC coupling was reduced. In other words, CSVD‐s patients mainly manifested as a disruption of the short‐range connections between adjacent areas.

Our latest studies found that changes in brain microstructure^41^ and functional topological networks^17^ were significantly associated with cognitive impairment in CSVD patients. In this study, our results indicated that SC‐FC coupling is significantly correlated with differentiated cognitive parameters for CSVD patients with different burdens. Specifically, SC‐FC coupling was positively correlated with MoCA and SDMT scores in the CSVD‐s group, which assessed mild cognitive impairment and information processing speed,^54^ respectively. The global/local efficiency of different CSVD burdens was positively correlated with the AVLT score, which assessed episodic verbal memory.^55^ We suspect that CSVD disrupts the linear relationship between structural and functional networks and information processing speed and visual memory. The cognitive performance results from the interaction between brain regions. We combined graph theory analysis to find that the damage characteristics of brain structure and functional network topology caused by different burden CSVD are different, which is related to cognitive performance. Our findings are analogous to the neurological and behavioral differences that occur in the development of normal aging, mild cognitive impairment, and Alzheimer's disease (AD). Studies have shown greater potential for reversal of cognitive deficits and enhancement of executive function during the MCI stage than during the AD stage.^56^ It may also explain the differentiated neurological‐behavioral correlations between CSVD‐s and CSVD‐m stages. The process by which the brain performs a task is represented by rapid changes in the recruitment and integration of brain regions that meet the demands of the task.^57^ For example, the motor module is recruited when performing the action, and the auditory module is recruited preferentially when we perform the auditory task. Notably, we found that modular SC‐FC coupling within the auditory/motor or limbic/subcortical module was associated with SCWT and TMT scores for CSVD patients, suggesting that the decline in attention, visual search, motor coordination, and information processing speed in CSVD is related to damage to the complex intrinsic neural network or “traffic pattern”^19^ in the brain.

This study has several limitations that should be noted. First, our current study did not explore the causal relationship between CSVD burden and the SC‐FC coupling value or functional network efficiency. Second, the cross‐sectional design of this study limits our ability to assess the dynamic evolution and disease progression of structural and functional networks. In our future research, we will strengthen the control of potential selection bias caused by parameters such as education levels and history of hypertension, the exploration of the causal relationship of CSVD, and increase the follow‐up of subjects to conduct a longitudinal investigation.

## 5 CONCLUSION

In summary, our study revealed that CSVD patients showed decreased whole‐brain and specific modular SC‐FC couplings. However, patients with different CSVD burdens exhibit distinct characteristics in network SC‐FC couplings, functional efficiency, and associations with cognitive dysfunction. Overall, the present study demonstrates that the impairment of SC‐FC coupling is related to the severity of CSVD and enriches the theoretical basis for evaluating its severity. Early detection of cognitive decline in patients with CSVD is crucial for improving and protecting their function and promoting the reversal of their cognitive deficits. The emergence of new biomarkers opens new avenues for better understanding and early intervention in this disorder.

## AUTHOR CONTRIBUTIONS

All authors participated in the recruitment of subjects. Xinyue Zhang and Changhu Liang wrote the initial draft. Na Wang, Yuanyuan Wang, Yian Gao, Chaofan Sui, Mengmeng Feng, Haotian Xin, and Nan Zhang collected the clinical data and imaging data. Hongwei Wen is mainly involved in the data analysis, design, and development of methodology, visualization of results, and guiding writing initial draft. Lingfei Guo and Hongwei Wen revised the main manuscript text. All authors reviewed the manuscript.

## FUNDING INFORMATION

This work was supported by grants from the National Natural Science Foundation of China (32100902, 82272072), the Fundamental Research Funds for the Central Universities (SWU118065), the Shandong Provincial Natural Science Foundation (ZR2020MH288), the Study Abroad Program by Shandong Province (201803059) and the Technology Development Plan of Jinan (201907052, 202328066). The authors thank all participants who participated in this study.

## CONFLICT OF INTEREST STATEMENT

The authors report no conflicts of interest.

## Supporting information


Data S1:

## Data Availability

The data that support the findings of this study are available from the corresponding author upon reasonable request.
