# Supplementary material for: Aberrant brain structural–functional connectivity coupling associated with cognitive dysfunction in different cerebral small vessel disease burdens
Source: CNS Neurosci Ther. 2024 Sep 3;30(9):e70005. doi: 10.1111/cns.70005 (PMC11371661; doi:10.1111/cns.70005)
Supplement: Supplementary file 1 — Data S1: [file CNS-30-e70005-s001.docx]

**Supplementary Materials**

**Supplementary Methods**

**Cognitive assessment process and criteria**

The TMT consists of two parts (TMT-A and TMT-B). The subjects were asked to connect randomly arranged circle numbers containing 1 to 25 in sequence as quickly as possible in TMT-A. TMT-B is similar to TMT-A, but subjects must alternate between numbers and letters. We used the time difference between completing the two tasks (TMT-B-TMT-A) as the judgment score. In SCWT, the subjects were asked to read three different forms as quickly as possible. Two of the tables represented "consistent conditions," in which subjects were asked to read the colors printed in black ink and the names of different color blocks. Instead, in the third table, the color word is printed with inks of inconsistent colors. The sum of time spent reading the three forms was used as the judgment score. In SDMT, the nine symbols correspond to the numbers 1-9. The lines to be answered contain only symbols, and the subject's task is to write or verbally report the correct number corresponding to the symbols in the following spaces. After completing the first 10 items under instruction, record how many responses the subject can complete in 90 seconds. The AVLT contains 12 words that are read three times in a row. Each reading was followed by a free recall test. Then, with a delay of 5 and 20 minutes, the subjects were asked to freely recall the 12 words again. Finally, recognition memory was tested using a 24-word disturbance list. The sum of the last six memorized words is used as the judging score.

**Structural network construction**

**Network node definition**: Individual T1-weighted images were coregistered to the b_0_ images in the DTI space. Then, the transformed T1 images were then nonlinearly transformed to the ICBM152 T1 template in the MNI space. The inverse transformations were used to warp the automated anatomical labeling (AAL) atlas [^1^](#_ENREF_1) from the MNI space to the DTI native space. Of note, the nearest-neighbor interpolation method was used to preserve discrete labeling values. Using this procedure, we obtained 90 cortical and subcortical regions (45 for each hemisphere), each representing a node in the network (Figure S1(1)-(3)).

**Network edge definition**: We performed probabilistic tractography for each subject (Figure S1(4)). Firstly, we used the bedpostx tool in FSL to run Markov Chain Monte Carlo sampling, in order to estimate distributions on diffusion parameters at each voxel, which allows to model crossing fibers within each voxel of the brain. Secondly, we used the probtrackx tool in FSL to perform probabilistic tracking. Briefly, we repetitively performed 5000 sampling from the distributions of voxel-wise principal diffusion directions, each time computing a streamline through these local samples to generate a probabilistic streamline fiber. For a seed region, 5000 × n streamline fibers were sampled; n is the number of voxels in the seed region. The number of streamline fibers passing through a given region divided by 5000 × n is calculated as the connectivity probability (P_ij_) from the seed region i to the given region j. To define the network edges, we computed w_ij_=P_ij_ as the weight between brain regions i and j. For each subject, a 90×90 structural connectivity (SC) weighted network/matrix was constructed (Figure S1(5)).

**
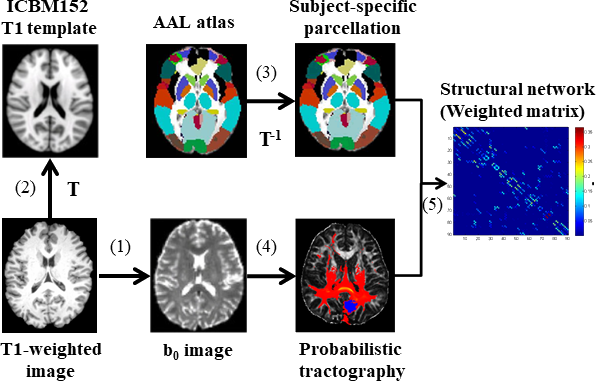
**

Figure S1. **Flowchart of structural network construction**. (1) Register the T1-weighted image to the b_0_ image in native space for each subject. (2) The transformed T1 images were then nonlinearly transformed to the ICBM152 T1 template in the MNI space, resulting in a nonlinear transformation (T). (3) Apply the inverse transformation (T^-1^) to the AAL atlas (90 regions) in the MNI space, resulting in a subject-specific parcellation of node regions in native space. (4) For each region, estimate the connectivity probability with other brain regions by using probabilistic diffusion [tractography](https://www.sciencedirect.com/topics/medicine-and-dentistry/tractography). (5) Construct the 90×90 weighted network for each subject by computing the connection probability between each pair of regions.

**Functional network topological metrics**

The shortest path length (L_p_) of network *G* are computed as follows:

$$L_{P}\left( G \right)=\frac{1}{N(N-1)}\sum_{i\neq j\in G} L_{\mathrm{ij}}$$

where L_ij_ is defined as the length of the path for node i and node j with the shortest length. For weighted networks, the length of each edge was assigned by computing the reciprocal of the edge weight (1/w_ij_).

The global efficiency of the parallel information transfer in the network, measured by the global efficiency (E_glob_) of network *G* [^2^](#_ENREF_2), can be calculated as follows:

$$E_{\mathrm{glob}}\left( G \right)=\frac{1}{N(N-1)}\sum_{i\neq j\in G} \frac{1}{L_{\mathrm{ij}}}$$

The local efficiency (E_loc_) of network *G* indicates how fault tolerant the network is and how efficient the communication is among the initial neighbors of node i when it is removed, which can be calculated as follows:

$$E_{\mathrm{loc}}\left( G \right)=\frac{1}{N}\sum_{i\in G} E_{\mathrm{glob}}(G_{i})$$

where G_i_ represents the subgraph that consists of the closest neighbors of node i.

To identify the regional/nodal topological properties of FC networks, we calculated the nodal efficiency E_nodal_(i), which measures the average L_p_ between a given node i and all of the other nodes in the network and is computed as follows:

$$E_{\mathrm{nodal}}\left( i \right)=\frac{1}{N-1}\sum_{i\neq j\in G} \frac{1}{L_{\mathrm{ij}}}$$

Table S1. Cortical and subcortical regions of interest and the corresponding functional modules defined in this study.

| Abbr. | Regions | Functional module |
| --- | --- | --- |
| PreCG.L | Precentral gyrus (Left) | auditory/motor |
| PreCG.R | Precentral gyrus (Right) | auditory/motor |
| SFGdor.L | Superior frontal gyrus, dorsolateral (Left) | DMN |
| SFGdor.R | Superior frontal gyrus, dorsolateral (Right) | DMN |
| ORBsup.L | Superior frontal gyrus, orbital part (Left) | attention |
| ORBsup.R | Superior frontal gyrus, orbital part (Right) | DMN |
| MFG.L | Middle frontal gyrus (Left) | attention |
| MFG.R | Middle frontal gyrus (Right) | attention |
| ORBmid.L | Middle frontal gyrus orbital part (Left) | attention |
| ORBmid.R | Middle frontal gyrus orbital part (Right) | attention |
| IFGoperc.L | Inferior frontal gyrus, opercular part (Left) | attention |
| IFGoperc.R | Inferior frontal gyrus, opercular part (Right) | attention |
| IFGtriang.L | Inferior frontal gyrus, triangular part (Left) | attention |
| IFGtriang.R | Inferior frontal gyrus, triangular part (Right) | attention |
| ORBinf.L | Inferior frontal gyrus, orbital part (Left) | attention |
| ORBinf.R | Inferior frontal gyrus, orbital part (Right) | attention |
| ROL.L | Rolandic operculum (Left) | auditory/motor |
| ROL.R | Rolandic operculum (Right) | auditory/motor |
| SMA.L | Supplementary motor area (Left) | attention |
| SMA.R | Supplementary motor area (Right) | auditory/motor |
| OLF.L | Olfactory cortex (Left) | Subcortical |
| OLF.R | Olfactory cortex (Right) | Subcortical |
| SFGmed.L | Superior frontal gyrus, medial (Left) | DMN |
| SFGmed.R | Superior frontal gyrus, medial (Right) | DMN |
| ORBsupmed.L | Superior frontal gyrus, medial orbital (Left) | DMN |
| ORBsupmed.R | Superior frontal gyrus, medial orbital (Right) | DMN |
| REC.L | Gyrus rectus (Left) | DMN |
| REC.R | Gyrus rectus (Right) | DMN |
| INS.L | Insula (Left) | auditory/motor |
| INS.R | Insula (Right) | auditory/motor |
| ACG.L | Anterior cingulate and paracingulate gyri (Left) | DMN |
| ACG.R | Anterior cingulate and paracingulate gyri (Right) | DMN |
| DCG.L | Median cingulate and paracingulate gyri (Left) | Subcortical |
| DCG.R | Median cingulate and paracingulate gyri (Right) | Subcortical |
| PCG.L | Posterior cingulate gyrus (Left) | DMN |
| PCG.R | Posterior cingulate gyrus (Right) | DMN |
| HIP.L | Hippocampus (Left) | Subcortical |
| HIP.R | Hippocampus (Right) | Subcortical |
| PHG.L | Parahippocampal gyrus (Left) | Subcortical |
| PHG.R | Parahippocampal gyrus (Right) | Subcortical |
| AMYG.L | Amygdala (Left) | Subcortical |
| AMYG.R | Amygdala (Right) | Subcortical |
| CAL.L | Calcarine fissure and surrounding cortex (Left) | vision |
| CAL.R | Calcarine fissure and surrounding cortex (Right) | vision |
| CUN.L | Cuneus (Left) | vision |
| CUN.R | Cuneus (Right) | vision |
| LING.L | Lingual gyrus (Left) | vision |
| LING.R | Lingual gyrus (Right) | vision |
| SOG.L | Superior occipital gyrus (Left) | vision |
| SOG.R | Superior occipital gyrus (Right) | vision |
| MOG.L | Middle occipital gyrus (Left) | vision |
| MOG.R | Middle occipital gyrus (Right) | vision |
| IOG.L | Inferior occipital gyrus (Left) | vision |
| IOG.R | Inferior occipital gyrus (Right) | vision |
| FFG.L | Fusiform gyrus (Left) | vision |
| FFG.R | Fusiform gyrus (Right) | vision |
| PoCG.L | Postcentral gyrus (Left) | auditory/motor |
| PoCG.R | Postcentral gyrus (Right) | auditory/motor |
| SPG.L | Superior parietal gyrus (Left) | auditory/motor |
| SPG.R | Superior parietal gyrus (Right) | auditory/motor |
| IPL.L | Inferior parietal, but supramarginal and angular gyri (Left) | attention |
| IPL.R | Inferior parietal, but supramarginal and angular gyri (Right) | attention |
| SMG.L | Supramarginal gyrus (Left) | auditory/motor |
| SMG.R | Supramarginal gyrus (Right) | auditory/motor |
| ANG.L | Angular gyrus (Left) | attention |
| ANG.R | Angular gyrus (Right) | attention |
| PCUN.L | Precuneus (Left) | DMN |
| PCUN.R | Precuneus (Right) | DMN |
| PCL.L | Paracentral lobule (Left) | auditory/motor |
| PCL.R | Paracentral lobule (Right) | auditory/motor |
| CAU.L | Caudate nucleus (Left) | Subcortical |
| CAU.R | Caudate nucleus (Right) | Subcortical |
| PUT.L | Lenticular nucleus, putamen (Left) | Subcortical |
| PUT.R | Lenticular nucleus, putamen (Right) | Subcortical |
| PAL.L | Lenticular nucleus, pallidum (Left) | Subcortical |
| PAL.R | Lenticular nucleus, pallidum (Right) | Subcortical |
| THA.L | Thalamus (Left) | Subcortical |
| THA.R | Thalamus (Right) | Subcortical |
| HES.L | Heschl gyrus (Left) | auditory/motor |
| HES.R | Heschl gyrus (Right) | auditory/motor |
| STG.L | Superior temporal gyrus (Left) | auditory/motor |
| STG.R | Superior temporal gyrus (Right) | auditory/motor |
| TPOsup.L | Temporal pole: superior temporal gyrus (Left) | attention |
| TPOsup.R | Temporal pole: superior temporal gyrus (Right) | auditory/motor |
| MTG.L | Middle temporal gyrus (Left) | DMN |
| MTG.R | Middle temporal gyrus (Right) | DMN |
| TPOmid.L | Temporal pole: middle temporal gyrus (Left) | Subcortical |
| TPOmid.R | Temporal pole: middle temporal gyrus (Right) | Subcortical |
| ITG.L | Inferior temporal gyrus (Left) | attention |
| ITG.R | Inferior temporal gyrus (Right) | DMN |

Note: The functional modular division of brain regions was based on a previous study [^3^](#_ENREF_3).

**Supplementary results**

**

**

Figure S2. The normal Q-Q (quantile-quantile) plots for education level of each group.

Table S2. The Kolmogorov-Smirnov (K-S) test results for head motion, age, education and cognitive test scores.

| Variable | group | Kolmogorov-Smirnov | | |
| --- | --- | --- | --- | --- |
|  |  | Statistic | df | Sig. |
| FD_Jenkinson | 1 | .084 | 54 | .200 |
|  | 2 | .080 | 106 | .106 |
|  | 3 | .095 | 79 | .185 |
| MoCA | 1 | .114 | 54 | .109 |
|  | 2 | .078 | 106 | .121 |
|  | 3 | .099 | 79 | .116 |
| AVLT | 1 | .108 | 54 | .191 |
|  | 2 | .077 | 106 | .128 |
|  | 3 | .084 | 79 | .200 |
| SDMT | 1 | .094 | 54 | .200 |
|  | 2 | .049 | 106 | .200 |
|  | 3 | .065 | 79 | .200 |
| SCWT | 1 | .109 | 54 | .187 |
|  | 2 | .086 | 106 | .063 |
|  | 3 | .101 | 79 | .083 |
| TMT(B-A) | 1 | .122 | 54 | .140 |
|  | 2 | .081 | 106 | .099 |
|  | 3 | .098 | 79 | .133 |
| age | 1 | .121 | 54 | .124 |
|  | 2 | .078 | 106 | .121 |
|  | 3 | .075 | 79 | .200 |
| education | 1 | .117 | 54 | .061 |
|  | 2 | .093 | 106 | .023 |
|  | 3 | .104 | 79 | .034 |

Note: group 1,2,3: CSVD-s, CSVD-m and control group.

Table S3. The Kolmogorov-Smirnov (K-S) test results for coupling/efficiency metrics.

| Variable | group | Kolmogorov-Smirnov | | |
| --- | --- | --- | --- | --- |
|  |  | Statistic | df | Sig. |
| $\mathrm{Coupling}_{whole brain}$ | 1 | .066 | 54 | .200 |
|  | 2 | .053 | 106 | .200 |
|  | 3 | .070 | 79 | .200 |
| $\mathrm{Coupling}_{auditory/motor}$ | 1 | .082 | 54 | .200 |
|  | 2 | .063 | 106 | .200 |
|  | 3 | .045 | 79 | .200 |
| $\mathrm{Coupling}_{\mathrm{vision}}$ | 1 | .075 | 54 | .200 |
|  | 2 | .055 | 106 | .200 |
|  | 3 | .075 | 79 | .200 |
| $\mathrm{Coupling}_{\mathrm{attention}}$ | 1 | .052 | 54 | .200 |
|  | 2 | .076 | 106 | .159 |
|  | 3 | .059 | 79 | .200 |
| $\mathrm{Coupling}_{\mathrm{DMN}}$ | 1 | .073 | 54 | .200 |
|  | 2 | .062 | 106 | .200 |
|  | 3 | .102 | 79 | .067 |
| $\mathrm{Coupling}_{\mathrm{subcortical}}$ | 1 | .057 | 54 | .200 |
|  | 2 | .057 | 106 | .200 |
|  | 3 | .081 | 79 | .200 |
| E_glob_ | 1 | .117 | 54 | .061 |
|  | 2 | .077 | 106 | .140 |
|  | 3 | .102 | 79 | .067 |
| E_loc_ | 1 | .116 | 54 | .077 |
|  | 2 | .064 | 106 | .200 |
|  | 3 | .089 | 79 | .189 |
| L_p_ | 1 | .090 | 54 | .200 |
|  | 2 | .071 | 106 | .200 |
|  | 3 | .065 | 79 | .200 |

Note: group 1,2,3: CSVD-s, CSVD-m and control group.


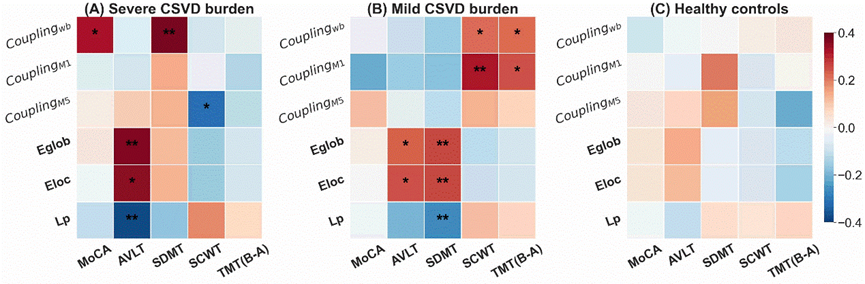


Figure S3. **Correlations between the coupling/efficiency metrics and cognitive scores in all groups**. Heatmaps of the partial correlation coefficients are shown for the (A) CSVD-s, (B) CSVD-s and (C) control groups. *: p<0.05, **: p<0.01; wb: whole brain; M1: auditory/motor module; M5: limbic/subcortical module.

Table S4. Descriptive statistical results for history of hypertension

| History of hypertension | CSVD-s | CSVD-m | HC |
| --- | --- | --- | --- |
| 0 | 13 | 54 | 51 |
| 1 | 40 | 52 | 27 |
| 2 | 1 | - | 1 |

Note: 0: No history of hypertension; 1: A history of hypertension; 2: Missing data, Note: CSVD-s: severe CSVD burden group (score≥2); CSVD-m: mild CSVD burden group (score≤1); HC: healthy control.

**Reference**

1. Tzourio-Mazoyer N, Landeau B, Papathanassiou D, et al. Automated anatomical labeling of activations in SPM using a macroscopic anatomical parcellation of the MNI MRI single-subject brain. *Neuroimage*. 2002;15(1):273-89.

2. Latora V, Marchiori M. Efficient Behavior of Small-World Networks. *Physical Review Letters*. 2001;87(19):198701.

3. He Y, Wang J, Wang L, et al. Uncovering intrinsic modular organization of spontaneous brain activity in humans. *PloS one*. 2009;4(4):e5226.
